# Amniotic fluid embolism: A rare cause of maternal collapse—A case report

**DOI:** 10.1002/ccr3.3433

**Published:** 2020-10-26

**Authors:** Sarita Sitaula, Dipti Das, Subhas Sitaula, Manisha Chhetry

**Affiliations:** ^1^ B.P. Koirala institute of Health Sciences Dharan Nepal; ^2^ Critical Care Unit Singing River Health System Pascagoula MS USA

**Keywords:** amniotic fluid embolism, coagulopathy, maternal mortality

## Abstract

High index of suspicion of amniotic fluid embolism should be considered in any intrapartum or postpartum collapse where the obvious cause of collapse is not identified.

## Graphical abstract

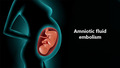

## 1 INTRODUCTION

We present a case of postpartum women who collapsed immediately after delivery with probable diagnosis of amniotic fluid embolism. This is a rare but fatal condition and was managed with supportive measures. Suspicion of amniotic fluid embolism should be considered in any postpartum collapse if no obvious cause is known.

Amniotic fluid embolism (AFE) is a rare complication of pregnancy, associated with significant morbidity and mortality, occurring in 2‐8 per 100 000 pregnancies.^1^ They usually present with sudden and unexplained cardiorespiratory collapse and disseminated intravascular coagulopathy.^2^ This is one of the causes of sudden death in obstetrics with a high mortality rate, ranging from 20% to 60%.^3^ Early recognition and prompt resuscitation are the key components for the management of AFE.

## 2 CASE PRESENTATION

A 30‐year‐old, unbooked primigravida at 39 weeks 6 days of period of gestation (POG) presented to obstetric emergency of BPKIHS, Nepal, referred from private hospital with diagnosis of intrauterine fetal death (IUFD) with obstetric cholestasis.

She had stopped perceiving fetal movements for 2 days. She gave a history of itching of the whole body, which had started at 35 weeks of gestation. Her liver function showed an increase in liver enzymes (SGPT/SGOT‐224/204IU/L, respectively), and the diagnosis of obstetric cholestasis was made clinically as bile acid concentration measurement was not available. She was started with tablet ursodeoxycholic acid 300 mg BD for 1 week, and then, she stopped the medication on her own.

Her 1st and 2nd trimesters were uneventful, and no other significant past and family history was present.

She is a nonsmoker and nonalcoholic.

### 2.1 On examination, at admission

She was well‐oriented to time, place, and person. Her vitals were within normal limits, with systolic blood pressure of 110 mm Hg and diastolic blood pressure of 70 mm Hg. Abdominal examination showed term size fetus in cephalic presentation without cardiac activity, and the patient was not in labor (Bishop score—3).

Her investigation reports at admission were all normal except increased liver enzyme values.

She was induced with 50 mcg of misoprostol, which was kept per vaginam 4 hourly on 2nd day of admission. She had progressed after 3rd dose of misoprostol and delivered a macerated female weighing 2.9 kg after 12 hours of induction. Labor and delivery were uneventful.

Around half an hour of the delivery, she had started having symptoms of hypoxia such as irritability, sweating, and anxiety. On examination, she was tachycardic and tachypneic with pulse rate of 170 beats per minute and respiratory rate of 24 breaths per minute, respectively. Oxygen saturation was up to 92% on room air. There was a drop in the blood pressure and became not recordable within a few minutes. The patient was conscious throughout with GCS of 15/15. The abdominal and local examination was normal, and there was no evidence of postpartum hemorrhage or ongoing blood loss. Resuscitation was started immediately with intravenous fluid and oxygen supplementation. Repeat blood investigations were sent, blood products were arranged, and the patient was shifted to ICU. After 1500 mL of fluid resuscitation, blood pressure was recorded up to 80/40 mm Hg, but there was no urine output. Arterial blood gas analysis showed lactic acidosis with metabolic alkalosis. The patient was started with noradrenaline as she was not maintaining blood pressure, which was continued for 36 hours and stopped gradually. The patient had started bleeding and soakage from the episiotomy site; hence, adrenaline packing was also done. Investigation reports were collected, which was suggestive of DIC. She was transfused with IV unit of fresh‐frozen plasma, IV units of fresh blood, and I unit of whole blood over 48 hours. Her renal function was deranged and creatinine values worsened in the first 3 days. However, as she had started passing urine after 8 hours of resuscitative measure, she did not require hemodialysis. She was also started with broad‐spectrum antibiotics and low molecular heparin, which was continued for 10 days.

The patient was improving after resuscitative measure but developed tachypnea on 4th postpartum day. A chest X‐ray was done and was suggestive of pleural effusion. Diagnostic pleural fluid tapping was performed, which was suggestive of transudative effusion. Echocardiography, D‐dimer, and APTT were normal, whereas electrocardiogram showed sinus tachycardia.

Multidisciplinary management of the patient was done in the maternal ICU for 8 days. She was then transferred to ward on 8th day as she was improving clinically and was discharged from hospital on 14th day. The patient was doing fine at 3rd week of follow‐up after discharge.

## 3 DISCUSSION

Amniotic fluid embolism is a rare complication but has a high fatality rate, characterized by sudden cardiovascular collapse, dyspnea, or respiratory collapse and disseminated intravascular coagulopathy.^4^ This may occur in healthy women during pregnancy, labor, or following delivery. It can develop even after elective abortion, amniocentesis, cesarean delivery, or trauma. This condition is initiated by entry of amniotic fluid to the bloodstream of the mother, which leads to a serious reaction causing cardiopulmonary arrest and massive coagulopathy.^3^, ^5^

Because AFE is a diagnosis of exclusion, a precise case definition and criteria are difficult to establish and also other causes of maternal collapse should be ruled out. The causes of maternal collapse could be due to hemorrhagic shock, pulmonary embolism, anaphylaxis, septic shock, and aortic dissection.^6^ Diagnosis is based upon the signs and symptoms observed during the birth or procedure. In this case, the patient had no history of high blood pressure and she was always normotensive. She did not have a history of fever, and the investigation report also supported to rule out sepsis. She did not have postpartum hemorrhage, and she was not anemic prior to delivery, which helped to rule out hemorrhagic shock.

The associated risk factors for AFE are age more than 35 years, multiparity, cesarean section, instrumental delivery, antepartum hemorrhage, eclampsia, labor induction, fetal distress, fetal death, and male baby.^7^, ^8^, ^9^

Few authors have proposed two clinical forms of AFE: typical and atypical. Typical or classic form has three phases: phase 1—respiratory and circulatory disorders, phase 2—coagulation disturbances, and phase 3—acute renal failure and acute respiratory distress syndrome (ARDS) leading to cardiopulmonary collapse.^10^ In atypical form, cardiopulmonary collapse does not occur, but the first symptom is life‐threatening hemorrhage due to DIC.^11^

The most significant diagnosis of AFE is made by findings at autopsy, which are limited to the lungs and clinical diagnostic criteria, and assisted by serum markers.^12^ Serum markers such as C3, C4, and C1 esterase inhibitors are reduced.

The symptoms are usually sudden in onset. Acute dyspnea, agitation, sudden chills, sweating coughing, and anxiety are common premonitory symptoms. Labored breathing and tachypnea may occur. Diagnosis can be made by the following criteria^2^, ^13^:

1. Acute hypotension or cardiac arrest
2. Acute hypoxia
3. Coagulopathy or severe hemorrhage in the absence of other explanations
4. All of these occurring during labor, cesarean delivery, dilation, and evacuation or within 30 minute postpartum with no other explanation of findings.

In our case, this woman had a risk factor for fetal death and labor induction. She did not have fever, obstetric hemorrhage, eclampsia, or any other identifiable cause of postpartum collapse, and also, she fitted to the abovementioned criteria of AFE; hence, we had made the diagnosis of AFE.

The initial phase of AFE consists mainly of right ventricular failure. If available, transthoracic and or transesophageal echocardiography may provide valuable information. Immediately after presentation, the echocardiography will reveal a severely dilated hypokinetic right ventricle (acute cor pulmonale) with deviation of the interventricular septum into the left ventricle. In our case, transthoracic echocardiography was done on 3rd day, which showed mild mitral regurgitation, mild pulmonary artery hypertension without any vegetation, and clots. As the patient was already stabilized, the finding may not be suggestive of AFE or pulmonary embolism. Also, early and aggressive resuscitation with blood product to correct coagulopathy results in improved outcomes.^14^

As computed tomography angiography or ventilation‐perfusion scan is not available in our center, we were not able to perform this test, which could have been performed to rule out pulmonary embolism. As this patient had coagulopathy, acute hypoxia and maternal collapse are in favor of AFE than pulmonary embolism.

Survival after AFE has improved significantly due to early recognition and management, and morbidity remains high with severe sequelae. Neurological impairment is the most common complication followed by renal failure, cardiac failure with left ventricular impairment, and arrhythmia have been reported.^8^

Primary management is respiratory support and hemodynamic support with judicious use of fluids, vasopressors, inotropes, and pulmonary vasodilators. Early diagnosis and aggressive management of patient with resuscitation improve the survival and long‐term morbidities.

## CONFLICT OF INTEREST

The authors declare that they have no conflict of interest regarding the publication of this case report.

## AUTHORS CONTRIBUTIONS

SS1 and DD: involved in diagnosing the patient. SS1 and DD: involved in the treatment and management of the patient. SS1: wrote the majority of the manuscript and formulated the hypothesis. SS2: helped and guided during patient diagnosis and management. MC: helped in formulating the manuscript. All co‐authors: provided critical feedback and helped shape the research, analysis, and manuscript.

## ETHICAL APPROVAL

As this was a case report, ethical approval from the Institutional Review Committee was not sought. However, written informed consent was obtained from the patient.

## PATIENT CONSENT

Written consent for publication was obtained from the patient.
